# Barriers and facilitators to engagement with between-session work for low-intensity Cognitive Behavioural Therapy (CBT)-based interventions: a qualitative exploration of practitioner perceptions

**DOI:** 10.1186/s12888-025-06501-3

**Published:** 2025-01-28

**Authors:** Mia Bennion, Amy Blakemore, Karina Lovell, Penny Bee

**Affiliations:** Division of Nursing, Midwifery and Social Work, The University of Manchester, Jean McFarlane Building, Oxford Road, Manchester, M13 9PL UK

**Keywords:** Cognitive behavioural therapy (CBT), Low-intensity interventions, Patient engagement, Between-session work (BSW), Qualitative

## Abstract

**Background:**

To address the growing demand for psychological treatment, healthcare providers are increasingly utilising low-intensity interventions, characterised by reduced practitioner contact and emphasis on independent patient engagement with therapeutic materials through between-session work (BSW). While BSW is critical for maximising treatment outcomes, patients and practitioners report challenges with its completion. Research identifying factors influencing between-session engagement in Cognitive Behavioural Therapy (CBT) has largely focused on high-intensity CBT, limiting understanding within low-intensity contexts.

**Methods:**

This study explored practitioner perspectives on the barriers and facilitators of BSW engagement within low-intensity CBT-based interventions. Using interpretive description methodology, 22 Psychological Well-being Practitioners (PWPs) from UK National Health Service (NHS) Talking Therapies services were interviewed. Inductive and deductive framework analysis was used to crossmatch data to an existing conceptual model of predictors for CBT BSW engagement.

**Results:**

Practitioners identified patient-level barriers including passive treatment expectations, comorbid health conditions, social stressors, and reduced mental health literacy, particularly among ethnic minority populations. Practitioner-level facilitators involved clear task planning and personalised BSW tailored to patients’ sociocultural environments. Organisational recommendations emphasised the need for diverse workforces and adequate training in culturally sensitive care.

**Conclusions:**

Findings underscore the importance of practitioner behaviours in optimising patient engagement between-sessions, offering clear directions to enhance treatment outcomes in globally adopted low-intensity interventions.

**Supplementary Information:**

The online version contains supplementary material available at 10.1186/s12888-025-06501-3.

## Background

Mental health problems, most commonly anxiety and depression, are a leading cause of disease burden globally, estimated to affect 970 million people [1]. The economic impact of mental health conditions is profound, with projected global losses amounting to US$16.3 trillion from 2011 to 2030 [2]. Despite these challenges, effective and cost-efficient interventions exist, with evaluations demonstrating the substantial economic benefits of scaling up treatment for depression and anxiety [3]. To this end, incorporating low-intensity interventions into service delivery models is a key strategy worldwide.

Heavily reliant on self-help materials or internet-based platforms, low-intensity interventions are brief, manualised treatments that can be delivered by non-specialists face-to-face, remotely or in group settings [4]. Evidence has demonstrated low-intensity interventions as effective in reducing symptoms of depression and anxiety in both high and low-and middle-income countries (LMICs) [5–8], offering a promising move towards universal health coverage. Whilst low-intensity interventions are considered the least intensive from a provider’s perspective, this is not intended to denote low engagement from the patient, with the success of treatment heavily contingent upon patient effort [9]. Patients are encouraged to take authority over their treatment, learning to self-manage difficulties by engaging with therapeutic activities during and between sessions.

Between-session work (BSW) broadly refers to activities which enable patients to collect information and apply new treatment insights and techniques to their everyday lives [10]. Tasks can involve psychoeducation or self-monitoring activities like thought diaries and the implementation of intervention-specific techniques such as worry postponement [11]. Within Cognitive Behavioural Therapy (CBT), systematic reviews and meta-analyses have shown positive associations between BSW engagement and treatment outcomes, with robust evidence of both causal [12] and correlational effects [13–15]. Within low-intensity interventions, between-session engagement has been perceived by patients as an important contributor to successful treatment outcomes [16] and has been shown to reduce depressive symptoms [17].

Issues with the completion of BSW are commonplace across all CBT interventions [18–20], including low-intensity models [17]. A previous systematic review sought to explore the predictors of engagement with BSW in CBT-based interventions and produced a combined theoretical and empirical model of patient engagement between-sessions, depicting multiple influences spanning patient, practitioner and environmental factors [21]. For example, patient beliefs regarding BSW and treatment such as perceived helpfulness, and practitioner competency in planning and reviewing BSW emerged as relatively consistent predictors of engagement with BSW. The impact of factors such as patient symptomology, sociocultural environment, practitioner beliefs and the therapeutic relationship were unclear, warranting further exploration. Notably, this review highlighted a significant gap in evidence regarding predictors of engagement with BSW within low-intensity contexts, emphasising a misalignment between the growing adoption of low-intensity approaches and the limited data supporting effective implementation.

Current initiatives exist to optimise engagement in low-intensity services such as the NHS Positive Practice guides which focus on improving experiences for underrepresented communities (i.e., 22), however there is no specific focus on BSW, leaving a critical gap in guidance and support. As global demand for mental health care continues to rise and resource-efficient, task-shifting methods become increasingly relied upon, the use and reach of low-intensity interventions are set to expand. Understanding the factors influencing between-session engagement in these settings is paramount to optimise provider training, guide research initiatives, minimise contextual inefficiencies, and maximise treatment outcomes. Frontline providers are uniquely positioned to provide valuable insights into the therapeutic processes influencing patient engagement with BSW, particularly given prior evidence synthesis highlighted practitioner strategies as key determinants of task engagement [21]. This study aimed to explore frontline provider perspectives of the barriers and facilitators of BSW engagement in low-intensity CBT-based interventions, assessing the applicability of the previously derived conceptual model [21] in a primary care low-intensity context.

## Methods

### Study design

This qualitative study was conducted within a pragmatist paradigm [23], employing an interpretive description methodology [24] which emphasises the utility of findings to enable practical consequences [25]. Participants were invited to participate in a single semi-structured interview, guided by a topic guide (Supplementary File 1) developed specifically for this study to elicit general attitudes to, and experiences of BSW, including perceived factors affecting between-session engagement.

### Ethical approval

The study was approved by the NHS North West Greater Manchester East Research Ethics Committee (Ref: 22/NW/0184).

### Sample and recruitment

Eligible participants comprised frontline practitioners working in UK NHS Talking Therapies services, namely Psychological Well-being Practitioners (PWPs), supervisors and trainee PWPs delivering low-intensity CBT-based treatment to patients at the time of the interview. This involved 30-minute individual treatment sessions delivered face-to-face, online, or via telephone. Purposeful sampling procedures were used to recruit participants with maximum variation in age, gender, ethnicity, and level of experience in mental health roles. Recruitment ceased when it was agreed within the research team that data saturation was achieved, defined as the point at which no new themes emerged during analysis [26].

Participants were recruited via participating Talking Therapies services in two NHS Trusts and one third sector organisation commissioned to deliver NHS services in the North-West of England. A ‘call for participants’ email was sent to low-intensity teams distributing study information packs containing a study invitation letter, Participation Information Sheet (PIS) and a consent-to-contact form. Interested participants who returned the consent-to-contact form and were contacted by MB using their preferred method. Active practitioners were also asked to share study information with relevant contacts to aid recruitment (snowball sampling). To address underrepresentation during recruitment, MB actively attended participating services’ team meetings, encouraging participation from groups underrepresented at the time, such as male practitioners and those from ethnic minority backgrounds. The study was also advertised on social media (X) and relevant newsletters/websites including University intranets to obtain geographical stretch of participants.

### Data collection and procedure

In accordance with ethical permissions, written or verbal informed consent was taken. Participation in the study was voluntary, with participants informed they could withdraw at any point prior to data analysis without providing a reason and without detriment to themselves. Once analysis commenced, withdrawal was no longer possible as data had been de-identified to ensure participant anonymity. This was clearly outlined in the PIS to ensure transparency. Participants were informed that interview data remained confidential unless concerns arose regarding participants’ safety or the safety of others. In such cases, a distress and/or risk protocol designed for the study would be activated where the researcher may be obligated to share information with the appropriate party, such as the participant’s GP or employer/professional body if evidence of misconduct/malpractice emerged. Data were securely stored on a University of Manchester server. Participants were offered a certificate to demonstrate their involvement in a qualitative research interview. Interviews were carried out between October 2022 - June 2023 at a time convenient for the participant. Participant preference determined interview modality, conducted either via telephone (*N* = 4) or online via Zoom/Microsoft Teams (*N* = 18). No participant requested a face-to-face interview. Interviews ranged from 40 to 118 min and were audio-recorded then transcribed verbatim by a university-approved transcribing service.

### Data analysis

Data management and analysis followed the five stages of framework analysis [27]. Transcripts were coded by MB, initially using an inductive approach where transcripts were read and re-read in full (Stage 1) and analysed using open coding techniques that assigned a data-derived code to meaningful segments. This was followed by a deductive approach where inductive codes were compared with a priori predictors of between-session engagement derived from the previous systematic review [21]. This process resulted in the development of a set of inductive and deductive themes, forming a working thematic framework (Stage 2). Data were then indexed and sorted using this framework into a matrix (Stage 3) and indexed data summarised during the charting stage (Stage 4). Finally, overarching or cross-cutting themes were identified (Stage 5). Throughout data analysis, preliminary codes and illustrative quotes were shared with the wider team (PB, KL and AB) who provided feedback on the proposed coding framework. Disagreements or alternative interpretations were resolved through discussion. Analysis was supported by QSR International’s NVivo-12 Plus qualitative software [28].

### Stakeholder engagement

During the design phase, feedback was obtained from an existing public and community engagement panel (*N* = 5) based in Greater Manchester. This panel included individuals from different socioeconomic and cultural backgrounds, including those with lived experience of health conditions and service use. During data collection, low-intensity staff from participating Talking Therapies services were consulted to identify barriers to participation and enhance recruitment strategies. Findings were then discussed with low-intensity teams (across two organisations) and a Lived Experience Advisory Panel (LEAP) established for this project, which included four individuals who had received low-intensity treatment. LEAP members were recruited with assistance from two Greater Manchester PPI coordinators who distributed opportunity forms to their networks. Applicants were screened and chosen based on their experience of receiving low-intensity CBT from a Talking Therapy service and were selected on a first-come, first-served basis. Feedback on data interpretation, practice implications, and knowledge mobilisation was collected from both stakeholder groups.

### Reflexivity

Members of the research team had both qualitative and clinical expertise with experience of delivering low-intensity interventions. During data collection and analysis, MB kept a research diary to reflect on potential bias and discussed this with the team. Two participants were known to MB via previous professional networks and one participant known via personal network. No other relationships between the interviewing researcher and participants were established prior to study commencement.

## Results

Invite reach could not be quantified as recruitment used open advertisement on social media. 27 individuals made contact, 25 were invited to interview and 22 consented and participated. Reasons for non-participation included no response following researcher reply to expression of interest (*n* = 3), ineligibility (*n* = 1) and study team consensus that data saturation had been met (*n* = 1). See Table 1 for participant characteristics.

**Table 1:** Participant characteristics

ID	Gender	Age group	Ethnicity	Current primary role	Time working in Talking Therapies services	Geographical area of employing service*
P1	Female	25–34	White British	PWP	1–5 years	South Yorkshire
P2	Female	45–54	White British	Senior PWP/supervisor	> 10 years	Cheshire/Merseyside
P3	Female	25–34	White British	PWP	1–5 years	Greater Manchester
P4	Female	25–34	White British	Trainee PWP	< 1 year	Greater Manchester
P5	Female	45–54	White and Black African	Senior PWP/supervisor	5–10 years	Cheshire
P6	Female	25–34	White British	Senior PWP/supervisor	5–10 years	Greater Manchester
P7	Female	25–34	White British	PWP	1–5 years	Greater Manchester
P8	Female	25–34	White British	PWP	1–5 years	Lancashire
P9	Male	25–34	White British	Senior PWP/supervisor	1–5 years	Greater Manchester
P10	Female	45–54	White British	PWP	5–10 years	Lancashire
P11	Female	25–34	Pakistani	PWP	1–5 years	Lancashire
P12	Male	25–34	Black African	Senior PWP/supervisor	5–10 years	Digital therapy provision across the UK
P13	Female	45–54	White British	PWP	> 10 years	Lancashire
P14	Female	25–34	White British	PWP	1–5 years	Lancashire
P15	Female	25–34	Pakistani	PWP	1–5 years	Lancashire
P16	Female	25–34	White British	PWP	1–5 years	Greater Manchester
P17	Female	25–34	Indian	Trainee PWP	< 1 year	Greater Manchester
P18	Female	25–34	White British	PWP	1–5 years	Northumberland
P19	Female	25–34	White British	Senior PWP/supervisor	1–5 years	Greater Manchester
P20	Female	25–34	Pakistani	PWP	1–5 years	Greater Manchester
P21	Male	45–54	White Welsh	PWP	5–10 years	Lancashire
P22	Female	45–54	White British	PWP	1–5 years	Buckinghamshire

*To maintain participant confidentiality, only the geographical area, rather than the specific service is provided.

### Role and engagement with BSW

Practitioners displayed positive attitudes towards BSW and reported consistent use of between-session activities in clinical practice. BSW was perceived as a key mechanism generating therapeutic change, optimising treatment outcomes and relapse prevention. Beyond the primary role of experiential learning, BSW was valued for enhancing psychological insight, normalising difficulties, and capturing useful data. BSW was also thought to sustain change due to technique retention and increased self-efficacy to self-manage difficulties. Although important in low- and high-intensity CBT, BSW was seen as crucial in low-intensity interventions due to limited ‘in-session’ time. Patient completion of BSW was also noted as personally rewarding for several practitioners, increasing job satisfaction.

“if we just do six sessions, half an hour long, we’ve had three hours of therapy, and I say, you coming in and talking to me for three hours won’t change much… it’s not enough to see long-term sustained changes. But if you do between-session work… this isn’t just three hours, this is 12 weeks of actual sustained behavioural change, and that is significant, that can change your life” (P9).

While practitioners placed great importance on BSW, they noted mixed patient responses, reporting common difficulties with completion which could signal treatment dropout. Practitioners appeared to dichotomise patients into those who do, and those who do not engage with tasks, placing less emphasis on the *quality* of BSW engagement. Generally, even for patients who were ostensibly engaging with BSW, practitioners felt engagement could be enhanced.

### Barriers and facilitators of between-session engagement

Three overarching themes, each with sub-themes, were constructed from the data, representing key change areas to enhance engagement with BSW: Managing expectations, Specificity, and Sociocultural environment (detailed further in Table 2).

**Table 2:** Perceived barriers and facilitators to between-session engagement across themes/sub-themes

Theme	Sub-theme	Barriers	Facilitators	Illustrative quotes
Managing expectations	Inclusion of between-session work	• View of therapy involving a passive rather than active approach	• Patient awareness and understanding of CBT-approach and guided self-help nature of treatment	“if people maybe assume that IAPT or low intensity CBT is a counselling approach or a notional support where you just come and offload and talk things through and just feel better then it probably comes as a bit of a shock if we’re setting something like a diary for them to go away and do after the first appointment.” (P1)
		• Negative views of between-session work/’homework’ stemming from educational experiences	• Early expectation management to allow patients to make an informed choice of the suitability of treatment	“I think the role that therapists play in that is really crucial and I think we don’t perhaps pay enough attention to that aspect. The groundwork, you know,…how are the therapists talking about in between-session work, the importance of homework, what are they spending their first sessions doing? Are they talking about it, are they identifying barriers?” (P12)
		• Negative previous experiences of CBT resulting in limited treatment expectancy and unhelpful pre-associations regarding between-session activities	• Practitioner emphasis on rationale for the inclusion of BSW	“I think homework, it can seem a little bit paternalistic, have you done your homework? So I do use the term between-session work… we’re not there to punish, we’re there to encourage.” (P9)
	Consequences of between-session engagement	• Unrealistic expectations regarding treatment progress i.e., techniques being a “quick fix”	• Realistic patient expectancies regarding the possible consequences of engaging with between-session activities	“some clients they might think CBT or guided self-help is a quick fix so, I think it is just explaining that it takes time and practice with a lot of clients anyway.” (P16)
		• Tasks with distal outcomes or anticipated negative consequences	• Tasks with proximal outcomes	“how they got on with it, did they have any difficulty with it, were they able to engage with it, how consistently were they able to engage with it during the two weeks between-sessions, what did they find of value with it, was there something they struggled with more, how did they feel that contributed to the improvement that we see, if there is any difficulty with that, it’s then reviewing what that difficulty might be and perhaps common pitfalls that sometimes might occur and mitigating approaches towards those.” (P21)
		• Excessive leniency and failure to address between-session disengagement	• Reassurances regarding the long-term rationale for between-session engagement	“I think there is that level of accountability that I’m sending you away with this sheet and you’re going to bring it back in two weeks filled out and we’re going to go through it together. Whereas over the phone, I’m saying, have you got that in front of you now? Can you see that? What have you written in it?” (P14)
		• Minimised accountability and increased challenges during the review of tasks within remote sessions	• Thorough practitioner review of BSW	
			• Productive practitioner response to non-completion of BSW and effective problem-solving of barriers to task completion	
	Professional expectations	• Rigid expectations regarding the pace of treatment and nature of BSW	• Reasonable expectations of between-session engagement with consideration of complexities	“From my experience of being a case manager, and a clinical skills supervisor, I feel that PWPs can sometimes feel the need to have some sense of completion; so I want them to be able to finish the course… Whereas my philosophy is, if it takes me six sessions with you for you to understand A, B, C… then that’s what we’re going to do.” (P9)
		• Lack of belief and enthusiasm for low intensity techniques sometimes exacerbated by practitioner burn out	• Clear expectations regarding patient-practitioner responsibilities throughout treatment	“practitioner beliefs, I feel like it plays a very important part. You know, seeing out of session work as something prescriptive, rather than seeing it as an added extra.” (P15)
		• Unproductive practitioner beliefs regarding BSW i.e., BSW as an optional ‘add-on’ to treatment		“even in that first conversation, I’ll emphasise that I can’t make you better, only you can make you better and that’s going to require effort from you, you are going to need to do these tasks.” (P4)
Specificity	Personalised care	• Practical obstacles related to task format i.e., online materials in non-edible formats or concerns regarding confidentiality when working with physical copies	• Personalised tasks aligned with patient capability, opportunity and motivation	“you can’t generalise, if you’ve met one ASD patient you’ve met one ASD patient. Well, you can say that about everybody and everyone so, it’s funny, why we don’t say that about everyone. If you’ve met one person, you’ve met one person.” (P13)
		• Limited availability of adapted materials	• Seeking patient preference regarding the format of materials	“we’re maybe just not a good fit with each other for whatever reason, and that relationship just isn’t there. Then sometimes it can feel a bit awkward in those sessions and maybe they’ve not got the belief in you or the belief in the therapy to actually want to go away and do those things.” (P14)
		• Practitioner assumptions regarding patient capability, opportunity and motivation	• Effective therapeutic relationship, strengthened further by showing efforts to tailor BSW	“women with endometriosis and that can then, if they do get their period and it’s the week of pain, they’ll come back and say I’ve spent the whole week in pain, I’ve just not even looked at it.” (P8)
		• Common depressive symptoms such as low motivation and energy impeding between-session engagement	• Adequate pacing and added flexibility to mitigate barriers arising from long-term health conditions i.e., pain and fatigue	
	Specific planning of BSW	• Vague task description and patient uncertainty regarding the task rationale	• Collaboration during task design	“when I was doing my training… I think I wasn’t explaining what to do enough, and then maybe they’ll get a little bit confused in between the sessions and were coming back and maybe half done the out [of] session work, I think it was because of me not being specific enough that hindered them.” (P3)
		• Signposting patients to between-session tasks without sufficient explanation or examples	• Personalised rationale for tasks aligned with patients’ goals	“I think that really sets them up for success by saying, right, when are you going to do this? And then they can go off and have a plan in their mind of what they’re going to do.” (P4)
			• Time management integrating BSW into patients’ schedules	“I’ll even say, when they start, say, something like BA… what happens if you plan to do this activity and it’s pouring with rain? It’s always good to have a plan B.” (P10)
			• Pre-empting barriers to completion with problem solving	
			• Gathering patient feedback on proposed tasks to assess sufficient procedural knowledge and opinion of BSW	
			• Written account of agreed BSW and an option for patients to get in touch between-session if required	
Sociocultural environment	Social context	• Social stressors i.e., housing instability and unemployment	• Greater socioeconomic status associated with perceived greater psychological insight	“For clients who come in and they say, no, it really is my priority to get better or to improve my mood, and it doesn’t matter how busy they are, they’ll still do it because they have dedicated themselves to it.” (P7)
		• Reduced social capital	• Prioritisation of between-session engagement alongside other responsibilities	“it’s just about allowing themselves that time to work on this because it can feel indulgent, but…it isn’t… if you can put this time in, then it’s going to mean not only are you well but…people around you might reap the benefit as well.” (P5)
		• Lack of time stemming from competing demands	• Signposting to external practical support to manage social stressors	“is now the right time, because I wouldn’t want you to carry on attending these sessions but not putting the in between session work into practice for whatever reason and have a negative benefit and not want to access help or the service in the future.” (P6)
			• Practical and emotional support provided by patients’ social network	
	Cultural context	• Limited mental health literacy and understanding of psychological treatment	• Option for patient to work with practitioner of similar ethnicity and culture to facilitate rapport and between-session engagement	“Ultimately, the question comes down to what the patient preference is. Is it, do I want someone who understands, I can save myself time in explaining things of how culture and religion works? Or do I want someone who’s completely oblivious to this and isn’t going to judge me for what I’m going to say?” (P11)
		• Referral prompted by others limiting motivation to engage between-sessions		“if I was told by my supervisors or my case manager… and they say you can try these things, I know they’ve worked in the past, there’s a little bit more transparency around this… people actually deviating from the structure a little bit, it would make practitioners a bit more competent in doing so as well.” (P20)
		• Dominating collectivist beliefs which potentially conflict with the perceived individualistic nature of BSW, exacerbated by societal gender roles		“there is an added layer of relying on the interpreter to explain things in the exact same way that you are, with the same level of compassion that you are, and the same level of openness that you are. So, you know, I might set homework with them, with the help of the interpreter, they might seem like they’re onboard with it, but when they come back to the session there might be a sense of, I didn’t quite understand what I was supposed to be doing.” (P15)
		• Dearth of culturally sensitive translated materials		
		• Perceptions of inadequate training and support to equip practitioner to deliver culturally sensitive care		
		• Reliance on interpreters to effectively mirror practitioner methods when integrating BSW in treatment		

### Managing expectations

#### Inclusion of BSW

Misconceptions of therapy and lack of awareness regarding CBT-based approaches perpetuated expectations of treatments akin to counselling with no BSW:

“we get letters from GPs saying this client needs counselling and they might be saying that when it isn’t really counselling, it’s CBT. So it’s a general term, they need talking therapy. They don’t necessarily know the difference…so people will come in with that expectation, there may not be any tasks or any between-session work.” (P5).

To mitigate this, practitioners expressed that early expectation management was critical. Providing a rationale for the inclusion of BSW in treatment was deemed vital to secure engagement, strengthened by explaining the patient’s active rather than passive role within treatment and clarifying the boundaries of practitioner responsibilities. Several practitioners described quantifying the importance of BSW using subjective estimates based on their experience:

“20%, 30% of your progress is what we do in therapy, but 80 per cent is what you do between our sessions, so it’s only going to work if you take part in between-session work.” (P20).

Practitioners viewed themselves as playing “a big part” in determining patient acceptance of BSW, related to how practitioners ‘sold’ tasks to patients including the language used. For instance, the term “homework” was thought to risk invoking potential negative perceptions formed from earlier school experiences and shape inaccurate judgements of patient and practitioner dynamics, depicting authoritarian rather than collaborative relationships. Previous experiences of CBT-based therapy were also reported to determine patients’ treatment expectations and could limit enthusiasm for BSW if strategies were deemed ineffective in the past, despite a host of possible contextual factors which may have led to previous treatment being unsuccessful.

#### Consequences of between-session engagement

Unrealistic expectations regarding treatment progress could hinder between-session engagement, particularly when patients held expectations of “quick fix” treatments. Once patients began to engage in activities, the consequences of doing so were thought to impact subsequent engagement. Some tasks were noted to have more obvious and immediate outcomes i.e., an instant reduction in anxiety, reinforcing engagement, whilst the rewards of other tasks i.e., thought challenging, could be less apparent and/or elicit negative consequences such as distress. When outcome expectancies such as improved mood were not met, this could leave patients questioning the utility of BSW, limiting engagement. Anticipated negative consequences, such as exposing oneself to fearful situations could also increase resistance to tasks. To prevent disengagement, practitioners described the need for transparency regarding possible consequences of BSW, alongside reassurances of the long-term distal benefits of engagement, achieving the overall goals for treatment.

The value of *thoroughly* reviewing BSW was emphasised, regardless of the extent of engagement, as an important method to acknowledge any new skills acquired and highlight positive change. Without this, patient efforts can feel devalued:

“If you are setting this between session work and then you’re not properly reviewing it… then they’re not going to feel like it’s an important part of the therapy, so they’re not really going to engage in it.” (P14).

Across the sample, practitioner responses to non-completion appeared to range from firm to lenient; stricter methods encompassed cancellation of the session if patients had not engaged with tasks whereas lenient approaches involved attempts to complete BSW in-session. Midway between the two were practitioners who explicitly addressed non-completion with patients, discussing barriers to completion and collaboratively problem-solving these to secure future engagement. Those applying stricter methods argued how the intervention cannot progress without the completion of tasks, emphasising that movement to the next stages of treatment was contingent on engagement with BSW:

“you got level one, you can’t get to level two until you’ve completed level one… we’re not moving on until this has been learned and acted on and integrated into your knowledge. Because otherwise we’re skipping to level six and you don’t have the skills to even do level two.” (P9).

Other practitioners feared patients feeling like “they’re being told off” or punished if tasks had not been completed, which could perpetuate difficulties and reduce engagement. By going ahead with the session, patients were still supported to understand how disengagement could hinder treatment progression as practitioners described how patients observed how incomplete tasks made the session feel less effective. The negative consequences of excessive leniency regarding non-completion were also reflected, highlighting the need to find an optimal balance:

“if I’m too flexible…it has started to become a habit where they’ll come back and then haven’t done the next thing… But I’ve thanked myself later on down the line, after I have been firm and then they’ve come back and they’ve done it, and then we can just move forwards rather than me - what’s the word? - jumping hoops for the clients, to try and think of something else that I can give them, when actually they haven’t engaged themselves in between.” (P7).

Whilst addressing disengagement was sometimes viewed as a “difficult conversation”, not leaving this as an “elephant in the room” was stressed. Both practitioners and supervisors reflected how response to non-completion can evolve as experience and confidence in the role are gained. Response was also thought to be moulded by organisational culture, stemming from supervision guidance and could vary depending on modality of treatment:

“because we used to work face-to-face more. If somebody came back and hadn’t done their between-session work, as a PWP I definitely would try and fill in the gaps and just revisit what they’ve done in the previous session…we’ve gone away from that a little bit now with telephone appointments especially, and maybe the way that we’ve heard other services do it. So, if somebody hasn’t done their between-session work that might mean the session doesn’t take place, because there’s nothing to discuss.” (P5).

While some practitioners noted no significant differences in between-session engagement across treatment delivery modalities, others found that reviewing tasks remotely posed challenges that negatively impacted engagement. They argued how face-to-face appointments facilitated better engagement, as patients were expected to bring worksheets for review. Similarly, in cases where patients could send completed documents before remote sessions (i.e., via secure email), engagement also improved. The inability to see completed worksheets during remote reviews meant practitioners relied solely on patient accounts to grasp the extent of engagement, which some felt diminished accountability and engagement:

“I sometimes think, you’ve not done the homework and you’re just saying that you have, but I can’t…I’m not calling you a liar, or anything like that, but you very quickly…get shoehorned in a different direction, and they’re trying to take you somewhere else” (P18).

#### Professional expectations

Practitioner expectations regarding BSW were identified to influence patient engagement with tasks. Participants discussed holding expectations of how between-session engagement ’should’ look throughout treatment and what stage of activities you ‘ought’ to have reached at a certain session, exacerbated by the structured nature and limited duration of low-intensity treatment. Such expectations could be adversely rigid, producing treatments moving at practitioners’ rather than patients’ pace and potentially hindering engagement if work felt overwhelming.

Favourable views of BSW and belief in low-intensity interventions was thought to promote patient engagement, helping patients to feel “inspired” to adopt techniques:

“I think you as a practitioner, if you’re not believing it, like, there is definitely such a thing as the contagion of beliefs, and not through any kind of mystical kind of process but just through how someone expresses the ideas that they have, and whether those ideas are taken on is really dependent on how that person… It’s hard to fake being enthusiastic about something” (P9).

Nevertheless, practitioners remarked such enthusiasm can be affected by the pressures of the PWP role or burnout. Practitioners noted how sometimes they too must remind themselves of the boundaries of their role as without maintaining BSW expectations, patients can edge into a passive approach, minimising responsibility and action between-sessions. Once more, this expectation was something practitioners felt more comfortable holding once more experienced:

“It’s supposed to be collaborative, it’s supposed to be engaging; it’s not supposed to be that you’re chasing someone to do something that they don’t want to do. So, I would only go so far in terms of encouraging, reminding, prompting. Whereas, like I said, maybe a few years ago I would have done a bit more to chase them up. But I’d find that that didn’t work, if anything it can be counterproductive.” (P2).

### Specificity

#### Personalised care

Personalising BSW to suit patients’ needs rather than a “one size fits all” approach was portrayed as a major facilitator to between-session engagement. Shared decision-making and developing rapport were voiced as key tools to personalise tasks which in turn fostered the therapeutic relationship and promoted between-session engagement further:

“I think the more you understand that person as well and they can see that you are understanding them and tailoring it to them… is one of the most important things for if they’re going to complete it or not” (P19).

The establishment of an effective therapeutic relationship was thought to facilitate BSW through several mechanisms: building trust, instilling belief in the practitioner and techniques, fostering honest communication regarding BSW, and promoting an aspect of shared power and mutual respect:

“clients who have engaged really well with homework, they’ve often attended sessions and said, you know, I couldn’t wait to tell you this and when I was doing it, I thought, you’d be really proud of me. And if there was no rapport, if there was no alliance, they wouldn’t probably say something like that” (P15).

Whilst facilitative of BSW, one practitioner noted how other aspects of the patient’s life can override the therapeutic relationship and impede BSW:

“I’ve got other clients that I’ve also got a great therapeutic relationship with but because of maybe their circumstances, for example, a perinatal client, they might struggle to do the in between-session tasks… so the therapeutic relationship is a factor I think but it’s definitely not like the deciding factor. It has some influence but not masses.” (P4).

Efforts to personalise BSW included aligning tasks with patient preferences and abilities, considering patients’ capability, opportunity and motivation. For example, individuals with dyslexia, ADHD, or low motivation may find certain resources overwhelming. Practitioners noted diversifying task formats to enhance engagement, using tools like guided relaxation audio for anxiety and psychoeducational videos (i.e., explaining the ‘fight, flight, or freeze’ response for panic treatment). The availability of adapted materials however, was perceived as limited and whilst practitioners commended resources like the NHS Positive Practice guides, one practitioner cautioned that such guidance might lead to reductionist viewpoints:

“when there’s a manual or guidance, people tend to adapt it or tend to use it in a literal sense, so this is the guidance therefore this will work for this person as opposed to this is the guidance, but I might still need to make additional adaptations for this person based on what they’re telling me” (P22).

Common reference was made to the patient’s presentation when personalising BSW. While some practitioners did not discern any patterns regarding the nature of the presenting problem and between-session engagement, there was a notion across the sample that those presenting with depression were less engaged between-session compared to those with anxiety presentations. This was attributed to symptoms such as low motivation, lack of concentration, and a “degree of hopelessness” hindering beliefs in techniques. Those who felt engagement with BSW was generally better for anxiety presentations listed dysfunctional motivators such as fear of negative approval or increased urgency spurred on by ‘what if’ worries. Conversely, several barriers stemming from anxiety were also mentioned, including avoidance and perfectionist tendencies.

Differing views were held regarding the influence of symptom severity on between-session engagement. Some felt that those with more severe symptoms were less engaged, particularly apparent with depression, due to the exaggerated symptoms impeding BSW. Here, to facilitate engagement, practitioners spoke of adjusting BSW, focusing on minimal but fundamental behavioural change such as eating or showering. Others spoke of positive influences stemming from severity, perhaps associated with readiness to change:

“… severe can be at that point where it is time for them to make that change and they just know that they need to do it and they know the time is now and they can be really, really motivated to do it, despite being quite impacted by their presentation.” (P21).

Practitioners appeared to distinguish severity from complexity, commenting whilst severity may not hinder BSW, complexity can, with common reference to comorbidities. Patients with a single mental health difficulty were perceived to engage better between-sessions than those with multiple challenges. Long-term physical health conditions (LTHCs) such as fibromyalgia and endometriosis were depicted to bring a host of barriers to BSW, including fatigue, chronic pain, and practical limitations such as reduced opportunity to complete work due to hospital stays or physical health appointments:

“If you set an activity with them where the patients in agreement, I might go for a five-minute walk but actually they’ve got so many physical health conditions, it’s like literally mission impossible for you to get up that day, it’s totally understandable you’re not going to get out of bed or go for that five-minute walk if you’re in that level of pain.” (P11).

Adjustments to BSW were again key to mitigate barriers, with most practitioners mentioning the use of adequate pacing and added flexibility. Service allowances such as additional sessions or fortnightly appointments was also thought to promote engagement.

#### Specific planning of BSW

Practitioners emphasised the importance of effective in-session planning of BSW to secure engagement. Specific planning involved several methods to provide patients with a clear understanding of *what*, *how*, and *why* they would engage in BSW. Whilst there is a heavy reliance on materials within low-intensity treatment, practitioners expressed concern about the effectiveness of simply directing patients to between-session resources without further discussion, questioning whether this provided sufficient support. Instead, talking through the activities, including working through examples, was thought to enhance engagement. Gauging patient understanding of the agreed BSW was deemed paramount to ensure patients possessed the procedural knowledge required to execute BSW as well as understanding the rationale. Action planning including allocating time to engage with BSW was perceived to be fruitful to ensure patients felt they had sufficient opportunity to engage with BSW, particularly when they had busy schedules. Seeking patient input to address barriers to completion was also thought to increase the likelihood of between-session engagement alongside providing written accounts of planned BSW which mitigated patient misunderstanding or forgetfulness. Practitioners discussed seeing differences in subsequent engagement if BSW was planned poorly:

“when I first started I just would send it over and give a brief explanation. That harboured pretty bad results because they would either not do the homework or they would do it incorrectly… So I now religiously always explain it in good detail.” (P17).

Whilst collaboration was an important factor for practitioners contributing to productive planning and between-session engagement, practitioners varied in their approach. Some assigned or recommended tasks gathering patient input regarding only how the task might be carried out, whilst others adopted a less prescriptive, patient-led approach, which involved encouraging the patient to devise the between-session activities themselves. Despite advocating for a Socratic approach to BSW, when working in half an hour windows, practitioners felt pushed to adopt more didactic methods.

### Sociocultural environment

#### Social context

Practitioners listed numerous social stressors which can limit BSW including housing instability, unemployment, and immigration processes. Experiencing a multitude of social issues can breed disengagement as patient attention is shifted away from BSW to addressing basic needs:

“if you think about it as like Maslow’s Hierarchy, your basic needs aren’t even being met. So self-actualisation, getting out and going for a jog, joining the gym, reading a book, like, living rather than surviving, you can’t get there because actually next door’s house has just been broken into, and your cousin’s been stabbed, or somebody’s had a drug overdose, or you can’t afford to pay the bills. All of this stuff is very much like the path needs clearing before you can start getting someone to sit down and do a diary.” (P9).

Some practitioners attributed higher socioeconomic status (SES) to greater psychological insight, underpinned by education level, which improved comprehension of techniques and between-session engagement. Greater social capital including both tangible (i.e., private space to complete BSW) and intangible resources (i.e., social support) were also thought to increase the likelihood of successful completion of BSW. Similarly, financial capital can allow patients to ‘buy’ time to engage with activities:

“if you’ve got someone who’s got a really comfortable job working from home, if they’ve got a husband, if they’ve got a carer who comes in or even a nanny who comes in to take care of the kids… they have that luxury of time, they can dedicate that time towards their selves and their wellbeing.” (P15).

Whilst lack of time was identified as a common barrier to between-session engagement, practitioners speculated how perceived lack of time may mask underlying barriers, such as insufficient readiness for change, where engaging with tasks is not prioritised. In cases where genuine time limitations or enduring social issues prevent engagement, practitioners considered whether signposting to other services for practical support or therapeutic approaches not involving BSW would be better suited. For some practitioners, the availability of time underpinned variations in between-session engagement across patient ages. These practitioners postulated greater engagement across older adults and more frequent disengagement across younger patients, where BSW risked being “just another thing to do” alongside educational homework. Conversely, others described how older populations expressed a preference to talk more in-sessions, perhaps exacerbated by feelings of loneliness, which lessened the focus on BSW. Younger patients were seen as more ‘socialised’ to completing tasks due to their educational activities which was alternatively felt to increase engagement.

#### Cultural context

The perceived impacts of cultural aspects to BSW were offered only by a select few participants, mostly from ethnic minority backgrounds. Practitioners described how individuals from ethnic minority populations can possess a limited understanding of therapy and psychological difficulties, generally encouraged by a family member or referred by their GP with minimal discussion of what treatment consists of. This can also make it hard for patients to explain their engagement with therapeutic activities to others in the community where dominating collectivist beliefs can conflict with the perceived individualistic nature of BSW, at times exacerbated by gendered expectations:

“I’m Pakistani and through my experiences and through working with people of the same background, it’s a very, sort of you know, collectivist culture, a sense of, the family unit and be there for other people as much as you want to be there for yourself. So, someone who has other responsibilities will be more likely to prioritise that rather than themselves, some people can come away feeling guilty for even sitting down and trying to block out all the other responsibilities that they have” (P15).

The advantages and disadvantages of pairing patients and practitioners with similar cultural backgrounds was discussed, seemingly impacting BSW through the ability to establish a productive therapeutic alliance:

“some patients do have a preference for somebody who’s from a similar background from them because you have that understanding about their culture, their religious views and they don’t have to explain themselves” (P11).

“this person might judge me because they’re of a similar background… people think, god, they might know the same people that I know, what if it gets back to my family and I’ve told them this? You know, I tell them the confidentiality policy so why would it pass…? I don’t know their family, but it is always that fear, I think, from them, that it could get out” (P17).

Practitioners described attempts to culturally adapt techniques to better suit patients yet questioned whether they were deviating from the intervention structure and best practice guidelines. Although culturally sensitive materials exist, practitioners expressed further expansion of these were required as well as training and adequate support to enable practitioners to integrate cultural considerations into treatment:

“I don’t think, as it stands between session work would conflict any religious values or cultural practices, but I think we can be a little bit more sensitive to those. So even though we’re not doing anything against them, we’re not really doing anything for them either… I used to have one client who, her religion was a massive factor so we were doing worry time and we included things like reciting the Quran or using the prayer beads or praying as one of our distraction techniques. I think that really helped her.” (P20).

Several practitioners spoke of various barriers to between-session engagement created when working with interpreters, particularly during remote treatment. Building a therapeutic relationship with patients was noted to be additionally challenging as the interpreter may change session-to-session and establishing rapport can depend on how effectively the interpreter mirrors practitioner methods. Given the influence of how BSW is portrayed, planned, and reviewed throughout therapy, practitioners expressed concerns of whether strategies can be lost in translation.

## Discussion

This study was the first to explore provider perspectives regarding the barriers and facilitators to engagement with BSW for low-intensity CBT-based interventions. Providers offered critical, experience-based insights, constructing three overarching themes pivotal to successful BSW. Themes signify the importance of practitioner behaviours to between-session engagement, mitigating against identified barriers, and displaying engagement-promoting strategies. Managing patient expectations regarding the inclusion, role and realities of BSW was deemed crucial to foster engagement. Specifically planned and tailored BSW to meet individual patients’ needs and preferences emerged as a crucial factor to ensure patients had the capability, opportunity, and motivation to complete tasks effectively. Additionally, factors rooted in patients’ sociocultural environment were acknowledged to influence the completion of BSW, encompassing barriers such as social stressors and familial obligations. Findings augment existing models of between-session engagement [21] and substantiate the applicability of model constructs to low-intensity contexts. The use of qualitative methods allowed for a deeper, more nuanced understanding of the factors influencing BSW engagement, uncovering novel concepts, particularly around expectation management and the sociocultural environment. Additionally, modifying factors were identified cutting across themes, such as treatment modality, practitioner job experience, and the therapeutic relationship.

Managing patient expectations regarding BSW from the outset, potentially during the referral or triage stage, was highlighted as a critical factor. Strategies may include educational materials that clarify the active nature of CBT and the pivotal role of BSW, addressing misconceptions that treatment is passive or a “quick fix” process. Practitioners can reinforce these expectations once treatment begins through direct discussion. Training and supervision should equip practitioners to communicate the value of BSW clearly and encourage consistent, thorough task review to acknowledge patient efforts and address challenges. Additionally, practitioners interviewed depicted how their own beliefs concerning low-intensity techniques can influence between-session engagement. This aligns with patient narratives regarding the factors impacting engagement with BSW, where patients described varying levels of practitioner integration of BSW during treatment, potentially underpinned by practitioner attitudes to BSW [29]. These findings emphasise the need to address both patient and practitioner attitudes to enhance engagement with between-session activities. Policymakers can support these efforts by embedding expectation management in national training programs, service protocols, and practitioner guidelines, ensuring a shared understanding of the value of BSW for achieving successful therapeutic outcomes.

Practitioners portrayed factors such as ethnicity, SES, comorbidity and cultural background to create distinct barriers to BSW, corroborating previous research [21, 30]. Such factors contribute to potential disparities in care where certain individuals are less likely, or less able to engage with BSW, thereby undermining equitable access to therapeutic benefits. Given that reducing mental health inequalities is a key priority for both NHS England [31] and global health initiatives [32], it is crucial to address the challenges faced by specific groups when engaging with between-session tasks. Practitioners should remain mindful of sociocultural determinants to BSW and their intersectionality, tailoring interventions accordingly to patients’ unique identities and circumstances.

While the earlier review model identified cultural concepts as a potential predictor of between-session engagement, the mechanisms behind these effects remained unclear [21]. The current study provides greater clarity, emphasising the need for organisational change to enable practitioners to deliver responsive treatment and tailored BSW. For instance, practitioners interviewed in this study and others [30] identified a significant gap in training and support to deliver culturally sensitive care, and limited availability of culturally appropriate, translated and adapted materials. Previous recommendations have called for the allowance of additional sessions with patients during treatment to give practitioners more time to understand cultural differences or adapt treatment for patients with LTHCs, emphasising the need for built-in service flexibility [33, 34]. For patients preferring to work with practitioners who share characteristics such as ethnicity or cultural background, the underrepresentation of ethnic minority practitioners within services constrains the potential benefits of such ‘matching,’ a factor already suggested to negatively affect engagement and treatment uptake [34]. However, some patients may not prefer to be matched by these characteristics, where concerns about confidentiality were cited by practitioners interviewed, highlighting the importance of enabling patient choice. Nonetheless, a more diverse workforce can facilitate knowledge transfer within services, with practitioners from minority ethnic backgrounds offering culturally specific insights and providing peer-led training to improve colleagues’ ability to culturally adapt interventions and BSW. Additionally, practitioners fluent in multiple languages could reduce reliance on interpreters, addressing challenges identified in this study, such as disrupted therapeutic continuity and strained rapport, which can hinder task engagement. By addressing these structural deficiencies, BSW represents a key avenue for achieving more inclusive care.

Ensuring responsive treatment is crucial for the effective implementation of low-intensity interventions within LMICs, requiring careful consideration of how, and for whom these treatments are designed to support. This involves adapting interventions to the local cultural and contextual realities, an aspect deemed to facilitate between-session engagement within this study. Such adaptations must go beyond simple resource translation, preserving the active therapeutic components to maintain intervention fidelity [35]. Although the need for local adaptation is emphasised, the role of BSW within this process is frequently overlooked and is rarely incorporated within cultural adaptation frameworks [36], highlighting a limitation in current models. While this issue is particularly relevant in LMICs, similar challenges exist in high-income countries (HICs). For example, some populations may seek religiously informed or culturally specific practices, highlighting the need for a more nuanced, context-driven approach to care.

Leveraging the adaptation of BSW offers a method to increase ‘buy-in’ from the population, which is often lacking [37]. Local actors, such as community health workers, trained to deliver the interventions are typically those who work closely with the population and possess valuable insight and expertise to develop locally relevant BSW. Moreover, community engagement, involving community leaders, traditional healers and people with lived experience of mental health conditions, is encouraged during intervention implementation and offers a further tool to personalise BSW to meet community needs. Given that BSW constitutes a significant portion of therapeutic activities in low-intensity interventions, it represents an underutilised tool for adapting interventions to diverse local contexts, ultimately enhancing the implementation and acceptability of global mental health solutions across both LMICs and HICs.

Remote delivery is a notable advantage of low-intensity treatments to enhance accessibility worldwide. The contemporary shift to remote work within low-intensity treatments [38, 39] and the current dominant prevalence of remote treatment in Talking Therapies services [40] is important to consider given practitioners described discrete barriers and facilitators to between-session engagement during remote sessions and indicated changing their practice according to modality. Practitioners recognised the need for adjustments to overcome barriers to BSW in remote sessions but emphasised the application of these are constrained by 30-minute session timeframes, balancing other requirements such as outcome measure review and risk assessment, reducing effective between-session engagement.

### Strengths and limitations

Several methods were adopted to enhance study validity including purposeful and maximum variation sampling, resulting in a sample with varied levels of practitioner experience from 13 diverse services across the UK. Both NHS and NHS-commissioned providers were represented, including a service providing purely digital support and a LTHC-specific service. Such diversity can increase the credibility of findings given shared patterns cutting across cases emerged out of heterogeneity [41]. Consultation with research users (i.e., patients/professionals) and the wider review team was sought throughout the research cycle to further enhance the study’s quality, relevance and impact. Findings resonated with staff contributors and LEAP members reinforcing the validity of data interpretation and ensuring outputs were meaningful for those who deliver and receive low-intensity treatment. Stakeholder consultation enabled the development of tailored knowledge mobilisation strategies, including patient-focused options involving animations, posters or leaflets to communicate findings, while professionals opted for training events addressing factors influencing between-session engagement. The representation of minority ethnic practitioners within this study, including those who deliver treatment in further languages, advocated for minority communities and provided similar views, substantiating arguments.

While the sample predominantly consisted of White British females (64%), this was lower than the 80% demographic reported in the current Talking Therapies workforce [42]. Despite employing various recruitment strategies, participants were self-selecting and aware of the research topic, which may have led to a higher representation of practitioners who were proponents of BSW. Furthermore, as the integration of BSW is expected every session as per low-intensity treatment protocols [43], social desirability may have deterred practitioners who less frequently use BSW in practice even though transcripts were anonymised. To minimise bias, the PIS noted that the study sought diverse perspectives on the topic, both positive and negative. Some participants were also aware the interviewer (MB) was a previous PWP which may have affected details provided in interviews. Although the research was situated within Talking Therapies services in the UK, rich descriptions and interpretations of the data allow for potential generalisation to other contexts [44].

## Conclusion

This paper offers a novel contribution to the field by employing qualitative methods to explore the barriers and facilitators of BSW for low-intensity interventions from the perspective of treatment providers. Although BSW is a core component of CBT-based approaches and one influential to treatment outcomes, participant narratives reported recurrent difficulties during BSW completion. Provider insights serve as an essential basis for systemic change by shedding light on modifiable factors that impact intervention effectiveness. Findings emphasise practitioner strategies as a fruitful avenue to enhance between-session engagement, capable of influencing patient and environmental factors impacting participation. Key areas for change centre around managing expectations concerning BSW, personalising and specifically planning tasks, and mitigating barriers originating from patients’ sociocultural environment. Organisational-level changes are also required, including greater diversity across practitioner workforces and more training, support, and resources to equip practitioners to provide culturally sensitive care. Failure to address the barriers highlighted by providers can limit engagement with BSW, perpetuate inequities in care delivery, and ultimately compromise treatment outcomes. Findings guide actionable improvements in practitioner training, service design, and policy support.

## Electronic supplementary material

Below is the link to the electronic supplementary material.

Supplementary Material 1

## Data Availability

The data that support the findings of this study are not publicly available due to ethical restrictions.

## Abbreviations

BSW: Between-session work
CBT: Cognitive Behavioural Therapy
PWPs: Psychological Well-being Practitioners
NHS: National Health Service
LMICs: Low-and middle-income countries
LEAP: Lived Experience Advisory Panel
LTHCs: Long-term physical health conditions
SES: Socioeconomic status
HICs: High-income countries
