# Supplementary material for: Barriers and facilitators to engagement with between-session work for low-intensity Cognitive Behavioural Therapy (CBT)-based interventions: a qualitative exploration of practitioner perceptions
Source: BMC Psychiatry. 2025 Jan 28;25:79. doi: 10.1186/s12888-025-06501-3 (PMC11776128; doi:10.1186/s12888-025-06501-3)
Supplement: Supplementary file 1 — Supplementary Material 1 [file 12888_2025_6501_MOESM1_ESM.docx]

**Supplementary File 1 - Interview topic guide**

**Definitions**

*What term(s) do you use to describe work completed as part of treatment between sessions?*

*What are some examples of activities completed between sessions?*

*If you were asked to describe what between-session work during mental health treatment was to someone unfamiliar with the term how would you describe it?*

**Attitudes**

*What is your opinion on between-session work? / How do you feel towards between-session work?*

*Do you feel between-session work is important in treatment?*

*What impacts do you think between-session work has/can have in treatment and beyond?*

**Experiences**

*Overall, how do you feel patients engage with between-session work?*

*Would you say engagement is consistent throughout the course of treatment or does engagement vary?*

**Factors affecting between-session engagement**

*What factors do you think can affect engagement with between-session work?*

*What can help engagement with between-session work?*

*What can hinder engagement with between-session work?*

*Can you think of anything which would help to improve client engagement with between-session work?*

*What do you find helps you assess barriers or facilitators when working with a client?*
